# *Brachycorynella asparagi* (Mordv.) Induced—Oxidative Stress and Antioxidative Defenses of *Asparagus officinalis* L.

**DOI:** 10.3390/ijms17101740

**Published:** 2016-10-20

**Authors:** Beata Borowiak-Sobkowiak, Agnieszka Woźniak, Waldemar Bednarski, Magda Formela, Sławomir Samardakiewicz, Iwona Morkunas

**Affiliations:** 1 Department of Entomology and Environmental Protection, Poznań University of Life Sciences, Dąbrowskiego 159, Poznań 60-594, Poland; borowiak@up.poznan.pl; 2 Department of Plant Physiology, Poznań University of Life Sciences, Wołyńska 35, Poznań 60-637, Poland; agnieszkam.wozniak@gmail.com (A.W.); formelamagda@o2.pl (M.F.); 3 Institute of Molecular Physics, Polish Academy of Sciences, Smoluchowskiego 17, Poznań 60-179, Poland; Waldemar.Bednarski@ifmpan.poznan.pl; 4 Laboratory of Electron and Confocal Microscopy, Faculty of Biology, Adam Mickiewicz University, Umultowska 89, Poznań 61-614, Poland; sas@amu.edu.pl

**Keywords:** *Brachycorynella asparagi*, *Asparagus officinalis*, semiquinone radicals, manganese ions, reactive oxygen species, oxidative stress, antioxidant enzymes

## Abstract

The aim of this study was to investigate whether and to what extent oxidative stress is induced in leaves of one- and two-month-old plants of *Asparagus officinalis* L. cv. Argenteuil infested by *Brachycorynella asparagi* (Mordvilko) at a varied population size. The pest *B. asparagi* has been described as the most damaging species feeding on asparagus. Analyses using electron paramagnetic resonance (EPR) demonstrated generally higher concentrations of semiquinone radicals with *g*-values of 2.0045 ± 0.0005 and 2.0026 ± 0.0005 in *Asparagus officinalis* (*A. officinalis*) leaves after *Brachycorynella asparagi* (*B. asparagi*) infestation than in the control. Observations of leaves under a confocal microscope showed a post-infestation enhanced generation of the superoxide anion radical (O_2_^•−^) and hydrogen peroxide (H_2_O_2_) in comparison to the control. Strong fluctuations in Mn^2+^ ion levels detected by EPR spectroscopy versus time were detected in leaves infested by aphids, which may indicate the involvement of these ions in the control of O_2_^•−^ production. An enhanced superoxide dismutase activity is an important element in leaf defense against oxidative stress. Visible symptoms were found in aphid-infested *A. officinalis*. Damage to leaves of one- and two-month-old *A. officinalis* plants by the aphid *B. asparagi* was dependent on the intensity, duration of infestation and plant age.

## 1. Introduction

Asparagus (*Asparagus officinalis*) is a perennial plant of the family Liliaceae. Since antiquity [1], asparagus has been cultivated as a vegetable crop for its succulent stalks (spears). Additionally, *Asparagus* was used in medicine (from which its species derived its name *officinalis*) for its diuretic properties [2]. China is the largest asparagus producer with almost 90% of the world production. In Europe, Spain is the first producer in the Mediterranean area, followed by Italy and France. Moreover, *A. officinalis* has also been cultivated in Poland for decades. *Asparagus* plants are colonized by a number of pests. Aphids, including the species *Brachycorynella*, infest aerial parts of the plant [3]. The European asparagus aphid, *Brachycorynella asparagi* (Mordvilko), is a major pest of garden asparagus. Feeding of the asparagus aphid causes a characteristic severe distortion of the terminal bud, called “rosetting” of *Asparagus* ferns, with the internodes being shortened and the leaves being both shortened and turning blue green. Strong aphid infestation on asparagus can cause dieback of seedlings [4]. Affected stems of mature plants are weakened; they develop imperfectly, become stunted and broom-like, with plants growing in the shape of a bush. Phytophagous insects represent a significant problem in global agriculture, causing yield reductions across all major agricultural areas and leading to costs incurred for control measures [5].

In the present study, we showed how an attack of *Brachycorynella asparagi* (*B.* *asparagi*) at a varied population size influences defense reactions, their intensity versus time of infestation, in leaves of one- and two-month-old *Asparagus officinalis* (*A. officinalis*) plants. Identification of defense responses in leaves of different *A. officinalis* ages in the context of the level of the oxidative stress and antioxidant defense against *B. asparagi* is a completely novel approach. The results of this study for the first time show the relative generation and the subcellular localization of reactive oxygen species (ROS) in *A. officinalis* leaves infested by *B. asparagi*. Superoxide anion radical (O_2_^•−^) and other ROS may be generated by cyclic reduction/oxidation of quinones, which may be reduced intracellularly to their semiquinones [6]. Moreover, post-infestation changes in concentrations of semiquinone radicals and manganese ions (Mn^2+^) in the above-mentioned system were revealed. Manganese, as a cofactor of superoxide dismutase (SOD) can participate in the plant′s defense against oxidative stress. In the available literature, there is practically no information regarding post-infestation changes of Mn^2+^ ions in plant response to aphid infestation.

The signs and symptoms of aphid attack can be diverse and vary depending on the plant species (and the tissue attacked), the aphid species and biotype, and their combination [7]. Plant survival upon herbivore attack depends on a multicomponent protection strategy, involving constitutive and inducible defenses [8,9]. The recognition of infestation activities by plants can occur through the use of transmembrane pattern recognition receptors (PRRs) or, acting largely inside the cell, polymorphic nucleotide-binding site-leucine-rich repeat (NBS-LRR) protein products, encoded by most resistant genes [10]. Recently new evidence for the involvement of PAMP (Pathogen Associated Molecular Pattern)-triggered immunity (PTI) in plant–aphid interactions was revealed by Jaouannet et al. [11]. Early plant responses to attacks by phytophagous insects or by pathogens share common events, such as protein phosphorylation, membrane depolarization, calcium influx and release of ROS, e.g., O_2_^•−^ and hydrogen peroxide (H_2_O_2_) [12]. Enhanced production of ROS or differential expression changes of genes involved in the oxidative stress in plants during feeding by aphids were found in several aphid–host systems [13,14,15,16]. An increased expression of respiratory burst oxidase homolog (RBOH), that plays an important role in ROS-mediated signaling, was also noted in aphid-infested leaves [17].

Amongst all, O_2_^•−^ and H_2_O_2_ are two ROS that have been given more importance in recent studies [18]. It has been established that H_2_O_2_ acts as a secondary messenger in signal transduction networks. Therefore, as it was reported by Maffei et al. [19,20], H_2_O_2_ is one of the most important ROS in the plant-pathogens/herbivore interactions and has an important role in signal transmission and in plant defense responses. Moreover, Mai et al. [16] reported that an early, strong generation of H_2_O_2_ may be related to its function as a signaling molecule, triggering defense mechanisms in pea leaves against *A. pisum*. Additionally, herbivore damage leads to the accumulation of secondary metabolites [21] and defensive proteins, including catalase (CAT), superoxide dismutase (SOD), peroxidase (POD), etc. [22]. As it was reported by Liang et al. [17], the expression pattern of some genes involved in the ROS scavenging system significantly changed after aphid infection. It has been widely reported that insect feeding influences the activities of plant defense enzymes and is associated with host resistance [23]. An unfavorable balance between ROS production and the plant antioxidant capacity seems to be responsible for the resulting susceptibility of the plant to insect attack [24]. The balance between ROS and antioxidant production determines the oxidative status of both plants and aphids, which in turn might influence aphids′ ability to infest new plants. Therefore, as it was reported by War et al. [25], the successful defense of plants against the biotic stresses depends on their ability to quickly perceive the incoming stimuli, decode them and build a strong morphological, physiological, and/or biochemical shield against the invaders. The oxidative state of the host plants is an important component of host plant resistance to insects [26,27,28,29].

The main objective of the present study was to examine whether and to what degree oxidative stress is induced in leaves of one- and two-month-old *Asparagus officinalis* L. cv. Argenteuil plants infested by *Brachycorynella asparagi* (Mordv.) at a varied population size (10, 20, 30 aphids per plant). Therefore, firstly we investigated the effects of *B. asparagi* on changes in the redox status of plants of cells, i.e., the concentration of semiquinone radicals and the levels of Mn^2+^ ions using electron paramagnetic resonance spectroscopy (EPR). The second goal was to determine relative generation and location of the superoxide anion radical and hydrogen peroxide in *A. officinalis* leaves infested by *B.* *asparagi*. Moreover, the third goal of the study was to determine superoxide dismutase activity, which is one of the most important antioxidative enzymes. It catalyzes the dismutation of the superoxide anion radical into hydrogen peroxide (H_2_O_2_) and molecular oxygen (O_2_). We also compared symptoms of *B. asparagi* infestation on *A. officinalis*, in this case one- and two-month-old *A. officinalis* L. cv. Argenteuil plants.

## 2. Results

### 2.1. Concentrations of Free Radicals and Mn^2+^ Ions Detected Using Electron Paramagnetic Resonance Spectroscopy in Asparagus officinalis Infested by Brachycorynella asparagi

**Figure 1:**
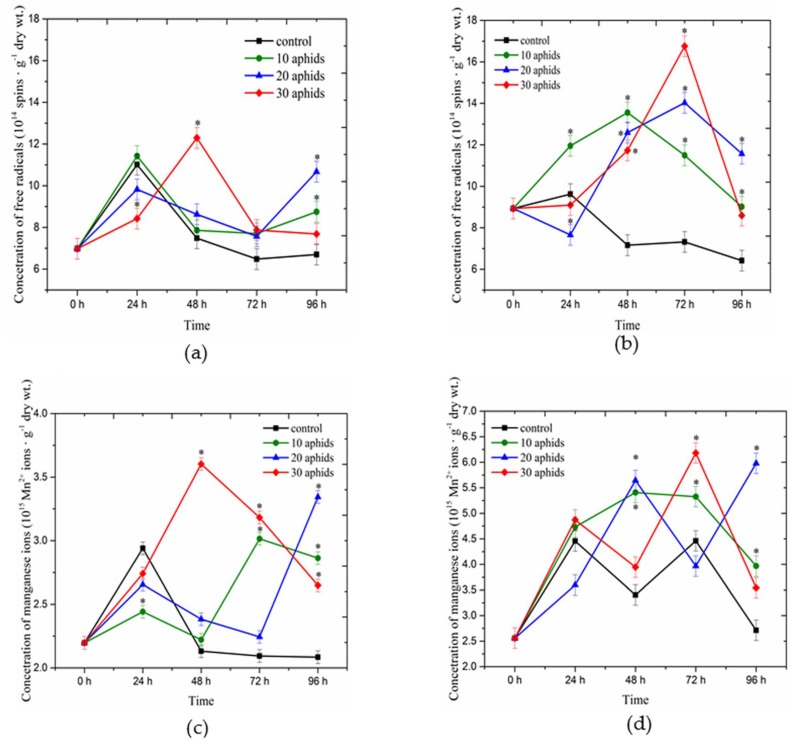
Concentrations of semiquinone radicals with two *g*-values: 2.0045 ± 0.0005 and 2.0026 ± 0.0005 (**a**,**b**); and manganese ions with *g*-values of 2.00 (0.01) (**c**,**d**) in leaves of one- and two-month-old plants of *Asparagus officinalis* L. cv. Argenteuil infested by *Brachycorynella asparagi* (Mordvilko) at a varied population size (10, 20, and 30 aphids per plant). Values represent means and SE from three independent experiments. The data was statistically analyzed using analysis of ANOVA variance (*p*-values at α = 0.05). In individual figures significant differences are shown using asterisks.

Analyses of electron paramagnetic resonance (EPR) revealed the presence of free radicals, which give signals with two *g*-values, 2.0045 ± 0.0005 and 2.0026 ± 0.0005, both in the non-infested and *B. asparagi* infested leaves of one- and two-month-old plants (Figure 1a,b), while signals of Mn^2+^ ions were recorded with *g*-values of 2.00 (0.01) in both types of these leaves (Figure 1c,d). Strong fluctuations were observed when analyzing changes in semiquinone radicals and in Mn^2+^ ions in the function of time in *A.* *officinalis* leaves infested by aphids and the controls. At the early stage of infestation (24 hpi) in leaves of one- and two-month-old plants infested by 20 and 30 aphids the level of semiquinone radicals was generally significantly lower in relation to the control (Figure 1a,b). In contrast, only in 24-h leaves of one- and two-month-old plants infested by 10 aphids the concentration of these radicals was higher than in the control plants; it was particularly visible in leaves of two-month-old plants. From 48 hpi after infestation the levels of these radicals were higher than in leaves not infested by aphids. The highest concentration of semiquinone radicals (12.29 × 10^14^ spins·g^−1^ dry wt.) was demonstrated in 48-h leaves of one-month-old plants infested by 30 aphids (Figure 1a), while in two-month-old *A.* *officinalis* leaves infested by 30 aphids the highest levels of these radicals (16.76 × 10^14^ spins·g^−1^ dry wt.) were recorded at 72 hpi (Figure 1b). In those infested leaves their concentrations were 1.5- and about 2.5-fold higher than in the control samples, respectively. Additionally, it is noticeable that in 72-h leaves of two-month-old plants infested by aphids the concentrations of semiquinone radicals were significantly higher in all infested variants than in the control plants and these levels were proportional to population sizes of aphids colonizing pea seedlings (Figure 1b). Moreover, when analyzing changes of these radicals in these leaves versus time it was observed that from 0 hpi up to 48 hpi their levels increased in leaves of one-month-old plants infested by 30 aphids (Figure 1a), followed by a drastic drop, although their level remained higher than in controls. In contrast, in leaves of one-month-old plants infested by 10 and 20 aphids we found a decrease in the level of semiquinone radicals from 24 to 72 hpi, after which at 96 hpi the level increased considerably and the concentration was higher than in the control plants. In turn, between 72 and 96 h after infestation a marked decrease was observed in the concentrations of semiquinone radicals in two-month-old *A. officinalis* leaves infested by aphids at a varied population size, with their levels remaining significantly higher than in the control (Figure 1b). Significant differences were observed between the applied experimental variants, i.e., leaves infested by aphids and the control, as revealed by ANOVA.

In turn, when analyzing changes in the level of Mn^2+^ ions it was found to decrease in 24-h leaves of one-month-old plants infested by aphids at a varied population size and in 24-h leaves of two-month-old plants infested by 20 aphids relative to the control (Figure 1c,d). Next, we need to stress a significant increase in Mn^2+^ ions in 48-h leaves of one-month-old plants infested by 30 aphids, as the level of these ions was 1.7 higher than in the control leaves. In turn, in the case of leaves of one-month-old plants infested by 10 aphids the highest concentration of these ions was recorded at 72 hpi. In the case of leaves infested by 20 aphids the highest concentration of Mn^2+^ ions was found at 96 hpi. Therefore, the levels of Mn^2+^ ions in those infested leaves were about 1.4 and 1.6 higher than in the control leaves, respectively (Figure 1c). ANOVA showed differences in these results to be highly significant. In leaves of one-month-old plants treated with 10, 20 and 30 aphids the levels of Mn^2+^ ions were (2.2–3.01) × 10^15^, (2.2–3.34) × 10^15^ and (2.2–3.6) spins·g^−1^ dry wt., respectively, while they ranged from 2.09 to 2.93 × 10^15^ spins·g^−1^ dry wt. in the control leaves (Figure 1c). In turn, in leaves of two-month-old plants infested by 10, 20 and 30 aphids the levels of Mn^2+^ ions were (2.55–5.40) × 10^15^, (2.55–5.97) × 10^15^ and (2.55–6.18) × 10^15^ spins·g^−1^ dry wt., respectively, while they ranged from 2.55 to 4.46 × 10^15^ spins·g^−1^ dry wt. in the control leaves (Figure 1d). It should be emphasized here that in leaves of two-month-old plants infested by aphids at a varied population size significant changes were observed in the function of time.

### 2.2. The Effect of Brachycorynella asparagi Infestation on the Generation of the Superoxide Anion Radical in Asparagus officinalis

**Figure 2:**
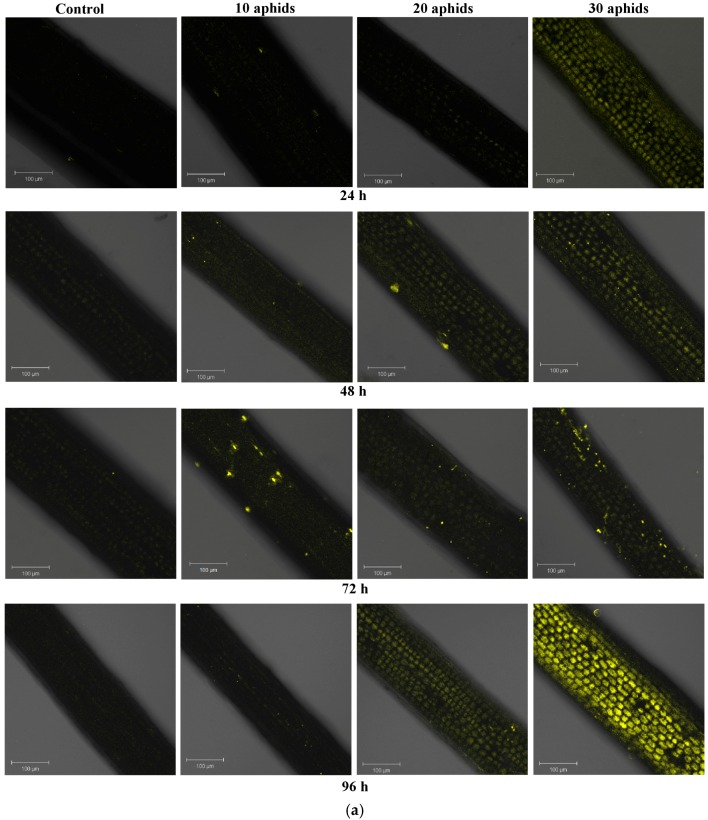

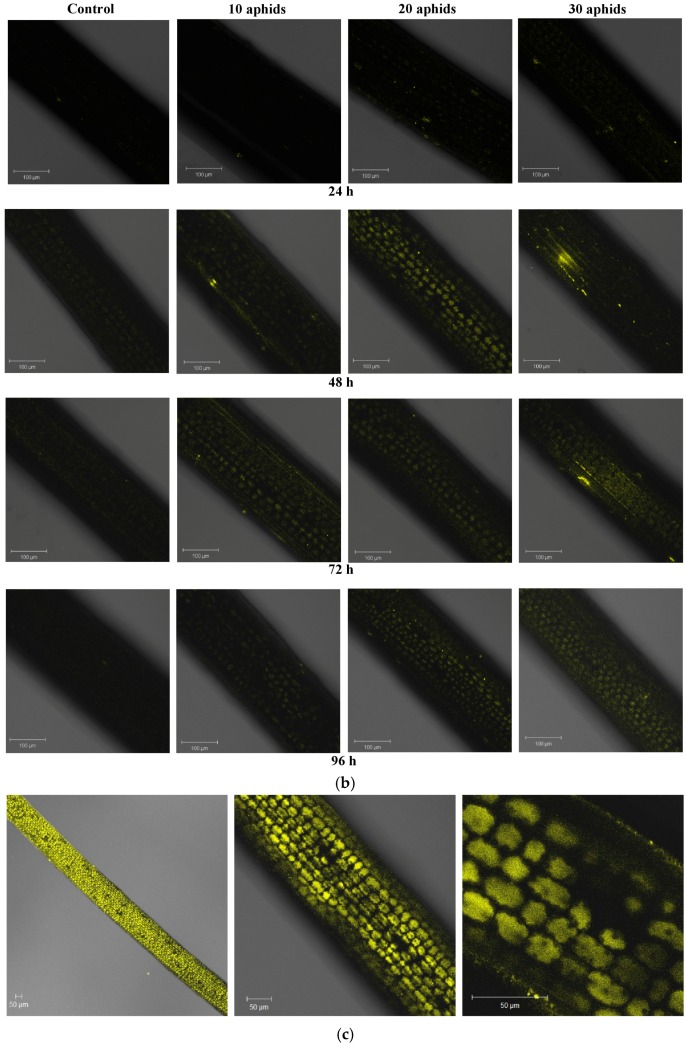
Relative generation and cytochemical localisation of superoxide anion radical in leaves of one- (**a**) and two-month-old plants (**b**) of *Asparagus officinalis* L. cv. Argenteuil infested by *Brachycorynella asparagi* (Mordv.) at a varied population size (10, 20, and 30 aphids per plant); and 96-h leaves of *Asparagus officinalis* one-month-old plants infested by 30 aphids *B. asparagi* per plant (**c**). Yellow fluorescence originating from DHE (dihydroethidium) was observed under a Zeiss LSM 510 confocal microscope (objective magnification of 20× for **a**,**b** and 5×, 20× and 63×, respectively, for **c**). Scale bar 100 µm (**a**,**b**) and 50 µm (**c**).

*Brachycorynella asparagi* infestation caused generation of the superoxide anion radical in *A. officinalis* leaves of both one- and two-month-old plants (Figure 2a–c). Relative release of O_2_^•−^ was investigated by staining leaves with the superoxide anion-specific indicator, dihydroethidium (DHE). The presence of O_2_^•−^ oxidizes DHE to ethidium, whereupon it emits fluorescence. On the surface of the leaves in the epidermal layer single points of O_2_^•−^ generation were visible. However, most of those places are visible on leaves infested by 20 and 30 aphids. Starting from 24 h in leaves infested by *B. asparagi* the production of O_2_^•−^ increased markedly with the duration of feeding time. The strongest generation was observed in 96-h leaves of one-month-old plants infested by 30 aphids. Additionally, it is noticeable that in these leaves infested by 30 aphids relative generation of O_2_^•−^ was significantly higher than in the control plants and in other infested variants (Figure 2a,c). Moreover, in 96-h leaves of one-month-old plants infested by 30 aphids the generation of O_2_^•−^ was visible mostly in the cytoplasm and sporadically in the regions of cell walls (Figure 2c). In turn, in leaf cells of two-month-old plants the generation of O_2_^•−^ increased markedly from 48 h (Figure 2b). In addition, relative generation of O_2_^•−^ was lower in leaves of two month-old *A. officinalis* plants than the leaves of one-month-old plants.

### 2.3. The Effect of Brachycorynella asparagi Infestation on the Level of Hydrogen Peroxide in Asparagus officinalis

**Figure 3:**
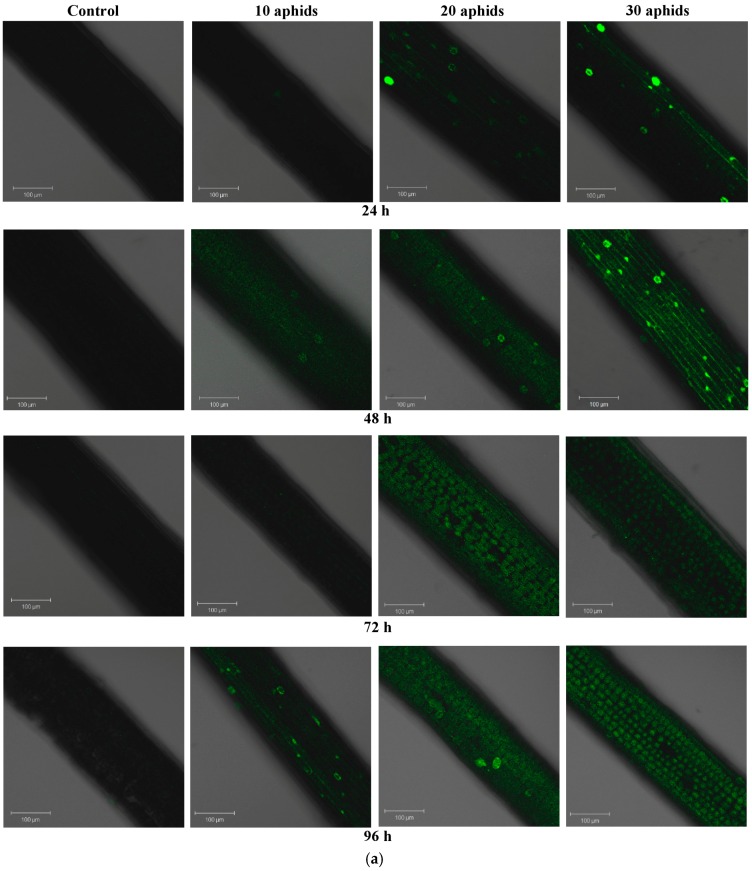

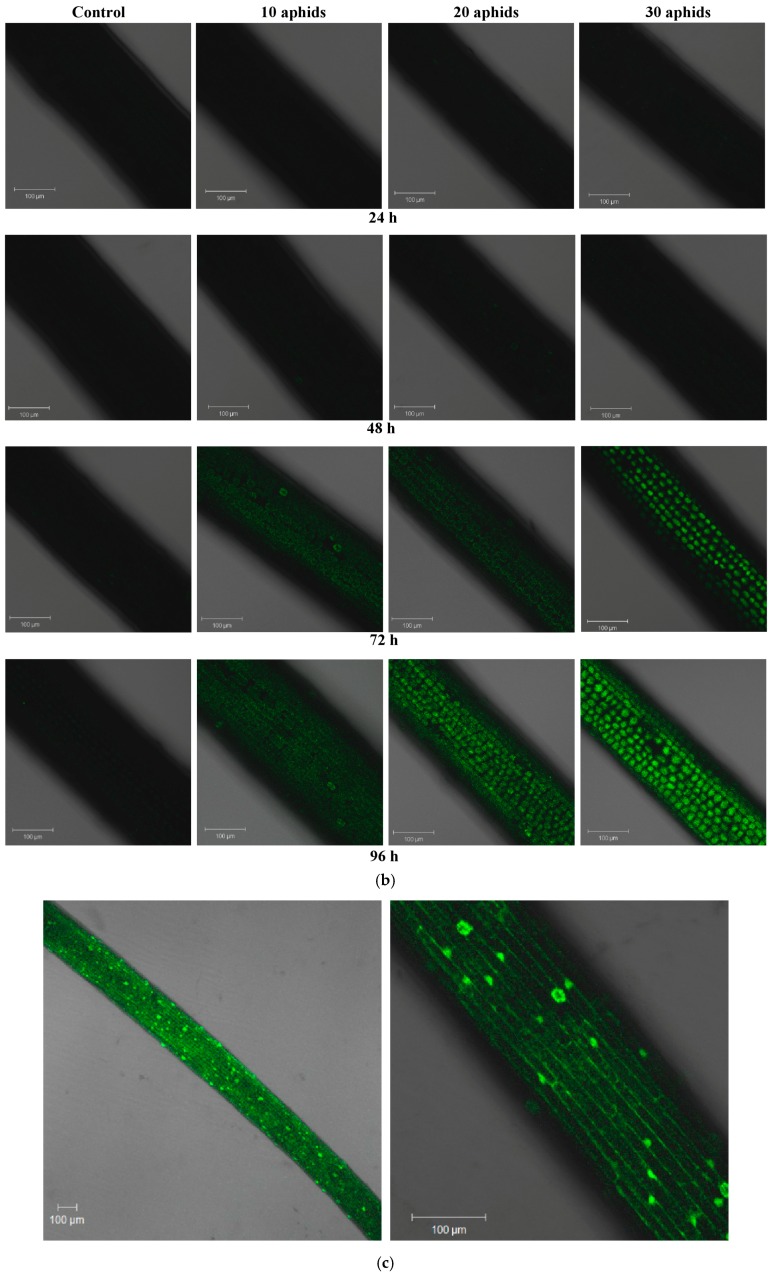

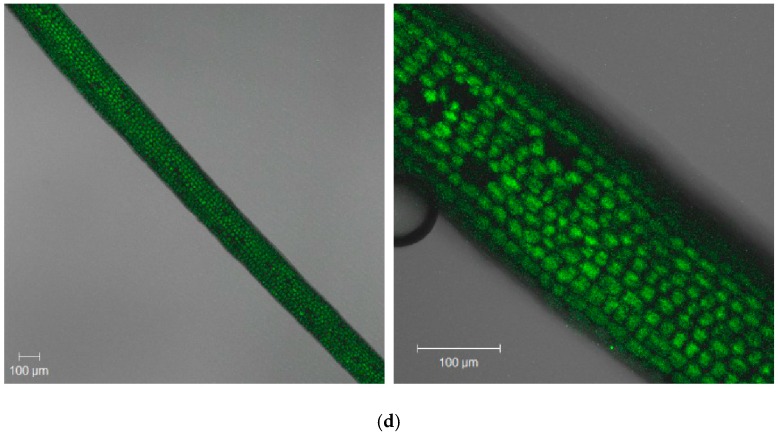
Relative generation and cytochemical localization of hydrogen peroxide in leaves of one- (**a**) and two-month-old plants (**b**) of *Asparagus officinalis* L. cv. Argenteuil infested by *Brachycorynella asparagi* (Mordv.) at a varied population size (10, 20, and 30 aphids per plant); 48-h leaves of *Asparagus officinalis* one-month-old plants infested by 30 aphids *B. asparagi* per plant (**c**); and 96-h leaves of *Asparagus officinalis* two-month-old plants infested by 30 aphids (**d**). Green fluorescence originating from DCFH-DA (dichlorodihydro-fluorescein diacetate) was observed under a Zeiss LSM 510 confocal microscope (objective magnification of 20× for **a**,**b** and 5× and 20×, respectively, for **c**). Scale bar 100 µm (**a**–**d**).

Already at 24 hpi in leaves of one-month-old plants infested by 20 and 30 aphids single spots of green fluorescence were visible, indicating H_2_O_2_ generation (Figure 3a). From 48 hpi we noted a stronger fluorescence in the function of time, which was visible in whole cells. However, in the early time of infestation of the pest *B. asparagi* the strongest generation of H_2_O_2_ was found in 48-h leaves of one-month-old plants infested by 30 aphids (Figure 3c). In turn, in 96-h leaves of one-month-old plants infested by 10 aphids the emission of green fluorescence was observed mainly in cell walls. Furthermore, *B. asparagi* feeding caused an increase in the generation of H_2_O_2_ in leaves of two-month-old plants infested by aphids, but it was noticeable starting from 72 hpi (Figure 3b). A strong emission of green fluorescence was observed in 72 and 96-h leaves of two-month-old plants infested by 30 aphids and in 96-h leaves infested by 20 aphids (Figure 3b,d). In these variants the emission of green fluorescence is most intense in the cytoplasm, while in 72-h leaves infested by 10 and 20 aphids and in 96-h leaves infested by 10 aphids this emission dominates in the area of cell walls.

### 2.4. The Effect of Brachycorynella asparagi Infestation on the Antioxidant Enzyme Activity in Asparagus officinalis

**Figure 4:**
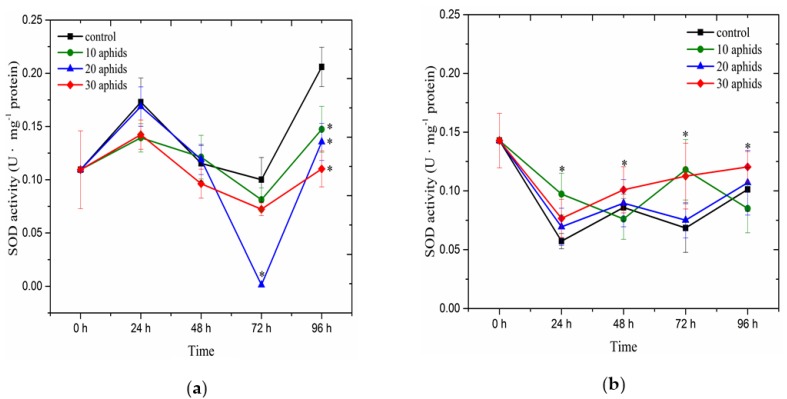
Superoxide dismutase activity in leaves of one- (**a**) and two-month-old plants (**b**) of *Asparagus officinalis* L. cv. Argenteuil infested by *Brachycorynella asparagi* (Mordv.) at a varied population size (10, 20, 30 aphids per plant). Values represent means and SE from three independent experiments. The data was statistically analyzed using ANOVA (*p*-values at α = 0.05). In individual figures significant differences are shown using asterisks.

Analysis of changes in the activity of SOD demonstrated a different trend in leaves of one- and two-month-old plants infested by aphids and the controls (Figure 4). In one-month-old plants of *A. officinalis* from 0 to 96 hpi we recorded a lower activity of this enzyme in the infested leaves than in the controls, with the exception of SOD activity in 24 and 48-h leaves infested by 20 aphids, wherein the activity of the enzyme was similar to that in the control (Figure 4a). At 72 and 96 hpi, we observed the largest reduction in the activity of the enzyme as compared to the control. In 72-h leaves infested by 10, 20 and 30 aphids, the activity of SOD was about 20%, 99% and 30% lower than in the control, respectively. In turn, in 96-h leaves infested by 10, 20 and 30 aphids, the activity of the enzyme was by about 28%, 34% and 46% lower than in the control, respectively. In contrast to SOD activity in leaves of one-month-old plants (Figure 4a), in two-month-old leaves infested by aphids the activity of SOD was higher than in the control plants, with the exception of SOD activity in 48 and 96-h leaves infested by 10 aphids (Figure 4b). In two-month-old leaves infested by 30 aphids the activity of this enzyme clearly increased from 24 to 96 hpi, while in the other experimental variants fluctuations were observed in the enzyme activity. At 48 and 96 hpi in leaves infested by 30 aphids the activity of SOD was highest in relation to the other experimental variants. In turn, at 96 hpi SOD activity in one-month-old leaves infested by 10 aphids was significantly lower than in the control. ANOVA showed differences in these results to be highly significant.

### 2.5. Symptoms of Brachycorynella asparagi Infestation on Asparagus officinalis

Considerable differences in the symptoms caused by *B. asparagi* were observed on *A. officinalis* one- and two-month-old plants (Table 1). These differences depended on the number of aphids on *A. officinalis* plants, the duration of infestation and plant age. Visible symptoms were found in aphid-infested *A. officinalis*, e.g., twisting of fern tops, shortening of internodes and yellowing of plants as well as growth inhibition. These symptoms were stronger on *A. officinalis* one- month-old plants infested by *B. asparagi* than two-month-old plants.

## 3. Discussion

The results of the present study revealed stress responses of *A. officinalis* L. cv. Argenteuil against *B. asparagi* concerning the level of oxidative stress and antioxidant defense dependent on plant age. The influence of age on the capacity of plants to mobilize a defense mechanism during aphid attack has not been discussed in the current literature. Our results indicate that younger, one-month-old plants induced faster defense responses to *B. asparagi* than older, two-month-old plants. However, in the early time of infestation of the pest *B. asparagi*, the generation of semiquinone radicals in leaves of one- and two-month-old plants demonstrated an increase, whereupon the response was different in those plants. Therefore, reduction of semiquinone radicals (being organic radicals) in 24-h leaves infested by 20 and 30 aphids was found in comparison with the control, which may indicate their incorporation into polymers, such as lignins, to protect the cell wall (Figure 1a,b). From 48 hpi levels of these radicals in infested leaves were significantly higher than in the control. The generation of semiquinone radicals from 48 hpi of feeding by *B. asparagi* is an important line of defense in *A. officinalis* leaves. The highest concentration of semiquinone radicals was observed in one-month-old *A. officinalis* leaves infested by 30 aphids at 48 hpi, while in two-month old leaves their highest generation was recorded at 72 hpi. Summarizing, these results show that in infested leaves of the younger plants the highest generation semiquinone radicals was found earlier than in older leaves. These radicals might have a cytotoxic impact towards *B. asparagi* and protect *A. officinalis* from damage or react with the O_2_^•−^, which relative generation in one-month-old plants infested by *B. asparagi* was higher than in the two-month-old plants infested by aphids. Moreover, earlier, higher generation of free radicals in one-month-old plants may also indicate their greater sensitivity to *B. asparagi*.

Semiquinone and phenoxyl radicals in plant cells are formed as a result of oxidation of hydroxyl groups in phenols and polyphenols [30]. The significant role of semiquinone radicals in defense of leguminous plants during an attack of biotic stressors was documented by Morkunas and co-workers also previously [31,32,33,34,35,36,37]. For example, concentrations of semiquinone radicals in *Acyrthosiphon pisum*-infested seedling leaves of *Pisum sativum* L. cv. Cysterski not only were generally higher than in the control plants, but also significantly increased versus time [16]. Besides, the strong generation and the continuous increase of O_2_^•−^ production in aphid-infested seedling leaves of pea from 0 to 96 h enhanced the defense potential to protect against *A. pisum*.

In this study, O_2_^•−^ and H_2_O_2_ relative generation was observed after *B. asparagi* infestation in confocal microscopy. Our results revealed post-infestation enhanced generation of O_2_^•−^ in *A. officinalis* leaves at all time points after infestation, in comparison to non-colonized plants (Figure 2a–c). Relative generation of O_2_^•−^ was lower in infested leaves of two month-old *A. officinalis* plants than infested leaves of one-month-old plants. In leaves of one-month-old plants most yellow fluorescence spots indicating generation of O_2_^•−^ were visible on leaves infested by 20 and 30 aphids. However, the strongest generation was observed in 96-h leaves of one-month-old plants infested by 30 aphids and was visible mostly in the cytoplasm and sporadically in the cell wall regions (Figure 2c). Recent reports indicate the involvement of ROS in a complex signaling network, which is necessary in the induction of defense reactions [38,39]. In addition, in the present study a stronger generation of H_2_O_2_ was observed earlier (at 24 hpi) in leaves of one-month-old plants than in leaves of two-month-old plants, especially in tissues infested by 30 aphids (Figure 3a–d). A strong emission of green fluorescence was observed both in cell walls and in the cytoplasm of 48-h cells of infested leaves (Figure 3c). In contrast to the generation of O_2_^•−^, a stronger emission of green fluorescence was observed in 72 and 96-h leaves of two-month-old plants infested by 30 aphids than in leaves of one-month-old plants infested by 30 aphids, being the most intense in the cytoplasm (Figure 3a–d). In addition, in our previous studies an early, strong generation of H_2_O_2_ at 24 hpi in pea aphid-infested leaves was observed, similarly as at 24 hpi in *A. officinalis* leaves of one-month-old plants infested by *B. asparagi*. ROS homeostasis is changed in response to stress; these compounds are regarded as molecules causing damage to cells at a high concentration, as well as ubiquitous signaling molecules at a low concentration, thus participating in recognizing and responding to stress factors [40,41]. Transduction of the signal modifies gene and protein expression levels, leading to physiological responses. ROS are produced in most plant subcellular locations, including the plasma membrane, mitochondria, nucleus, chloroplast and the cell wall [22].

Moreover, in this paper we provide evidence using EPR spectroscopy for strong fluctuations in Mn^2+^ ion levels versus time in *A. officinalis* leaves infested by aphids at a varied population size (Figure 1c,d). At 24 hpi the concentration of these ions in one-month-old plants infested by aphids (for all aphid population sizes) was lower than in the control plants (Figure 1c), while in leaves of two-month-old plants infested by 20 aphids it was lower only in relation to the control leaves (Figure 1d). These results may indicate the involvement of these ions in the control of O_2_^•−^ production. From 48 hpi, the concentration of Mn^2+^ ions was generally higher in infested leaves than in the control. As it was reported by Lidon et al. [42], manganese might control the production of O_2_^•−^ (as an isolated ion or as a cofactor of SODs) and directly participate in oxidation processes. Besides, manganese found in different redox states performs different functions. Shenker et al. [43] reported that manganese is involved in both oxygen radical detoxification via its role in SOD activity, and oxygen radical production via its involvement in the photosynthetic pathway. Besides, manganese is also essential for the biosynthesis of chlorophyll (through the activation of specific enzymes), aromatic amino acids (tyrosine) and secondary products, such as lignin and flavonoids [42]. However, it is remarkable in our study that the activity of SOD in one-month-old plants infested by aphids (for all aphid populations) was lower than in the control plants during this experiment (Figure 4a). Perhaps this is related with the maintenance of O_2_^•−^ generation at a certain level, which is an important element in the defense strategy of these plants against *B. asparagi*. In contrast, in two-month-old plants infested by aphids the activity of SOD was higher than in the control plants (Figure 4b). In parallel, in these tissues Mn^2+^ ion levels were higher than in the control plants. An enhanced SOD activity can be an important element in the defense against oxidative stress of two-month-old plants infested by aphids. Analyses of symptoms of *B. asparagi* infestation on *Asparagus officinalis* demonstrated that two-month-old plants were less infested than one-month-old plants (Table 1). To prevent ROS toxicity, organisms have evolved antioxidant enzymes, including SOD [44], which activity was studied in this work. Besides, War et al. [25] showed that induction of enzyme activities and the amounts of secondary metabolites were greater in the insect-resistant groundnut genotypes ICGV 86699, ICGV 86031, ICG 2271, and ICG 697 infested with *H. armigera* and *A. craccivora* than in the susceptible check JL 24. SOD catalyses the conversion of superoxide radicals to the more stable H_2_O_2_ that is required for long-range cell-to-cell signaling or for passing through membranes [45]. Additionally, SOD represented the first line of defense against ROS [46]. Moreover, Kerchev et al. [15] found that the gene encoding a putative cytosolic Cu/ZnSOD was upregulated in *Myzus persicae*-infested potato plants, whereas the levels of an FeSOD gene transcript gradually declined. It has been suggested that genes involved in oxidative stress are key components of plants infested response [47,48,49]. In addition, the results obtained by Sytykiewicz [50] suggest that the greater SOD response of seedlings of *Zea mays* L. Ambrozja (a relatively resistant cultivar) relative to cv. Tasty Sweet (susceptible) seedlings might be an important factor in the ability to alleviate the aphid stimulated oxidative burst and thus may be the basis of Ambrozja’s greater ability to survive infestation. Moreover, it was reported that pea aphid colonization led to a significant insect density- and time-dependent enhancement in the rate of H_2_O_2_ and O_2_^•−^ production in pea seedlings [16]. Moreover, it was shown that *Aphis craccivora* at a varied population size (10, 20 and 30 individuals per each soybean plant) caused a burst of H_2_O_2_ generation in the aphid-infested leaves at 12 hpi. Paralleling the H_2_O_2_ accumulation, peroxidase activity in all the infested plants remarkably increased and was significantly higher than that observed in the controls [51]. In turn, Ren et al. [52] observed an H_2_O_2_ flux in mesophyll cells versus time for tobacco samples with different infestation rates of *Myzus persicae* treatment. Additionally, studies performed by Sytykiewicz [50] evidenced profound intervarietal differences in the accumulation of H_2_O_2_ and relative expression of two catalase genes in maize seedlings challenged by cereal aphids′ herbivory.

It should be mentioned here that characteristics of the plant, including organ or tissue identity, developmental stage and genotype, also influence plant response to stress [53]. At specific developmental stages, plants are either more or less sensitive to stressors. The sensitivity stages of development are called windows of sensitivity [54]. Additionally, different adaptive mechanisms can be observed, which depend on the species considered, the organ, the tissue, the age and the exposure intensity of the stress [40]. Moreover, Donovan et al. [55] reported that during plant life stages the levels of plant hormones fluctuate, which affects the induction of defense mechanisms. Besides, different defensive strategies are activated in response to different modes of feeding by insects.

## 4. Materials and Methods

### 4.1. Plant Material

All experiments were performed on *Asparagus officinalis* L. cv. Argenteuil, which seeds were provided by the PlantiCo Company in Zielonki near Warszawa (Poland). Asparagus seeds were imbibed in sterile water in an incubator at 23 °C. After 24 h of imbibition the seeds were transferred to Petri dishes and immersed in a small amount of water in order to support further absorption. After a subsequent 48 h imbibed seeds were sown in pots of 20 cm in diameter containing sterilized soil (12 seeds to the pot). One- and two-month-old plants were kept in the growth chamber at 22–23 °C, 65% relative humidity and light intensity of 130–150 µmole photons·m^−2^·s^−1^ with the 14/10 h (light/dark) photoperiod.

The experimental materials, i.e., leaves of one- and two-month-old *A. officinalis*, were carefully collected at 0, 24, 48, 72 and 96 h post infestation (hpi). Leaves were weighed and then were frozen in liquid nitrogen for subsequent analyses of semiquinone radicals, Mn^2+^ ions and the activity of superoxide dismutase. Analyses of the generation of hydrogen peroxide and the superoxide anion radical were performed in fresh materials at particular time points for all the variants.

### 4.2. Aphids and the Infestation Experiment

*Brachycorynella asparagi* (*Mordv*.) (Mordvilko 1929) (Hemiptera: Aphidoidea) were cultured and supplied by the Department of Entomology and Environmental Protection, the Poznań University of Life Sciences. They were reared on its host, *Asparagus officinalis*, in the growth chamber at the Department of Plant Physiology, the Poznań University of Life Sciences at a temperature of 21–22 °C, 65% humidity and the photoperiod of 14/10 h (light/dark) with light intensity of 130–150 µM photons·m^−2^·s^−1^.

All plants were infested with 10, 20 and 30 apterous adult females of *B. asparagi*. Each variant had 4 pots in total. In the experiments one- and two-month-old plants were used. This developmental stage proved to be the most suitable for *B. asparagi* aphids, which was demonstrated by high daily fecundity (Sobkowiak, unpublished data). Development of aphids is dependent on the age of the plant; generally, aphids better infest developmentally younger plants with high turgor rather than developmentally mature plants. Aphid specimens were carefully transferred to leaves and twigs with a fine paintbrush. The aphid populations were monitored throughout all the experiments. The number of apterous females of *B. asparagi* remained constant in each experiment, because newly born larvae were removed once a day. The control plants in the experiments were one- and two-month-old plants with no aphids. All the control and aphid-infested plants were put separately into glass boxes (30 cm × 28 cm × 30 cm) covered with nylon gauze and placed in a growth chamber with the environmental factors controlled.

### 4.3. Electron Paramagnetic Resonance (EPR)

Samples of 1 g fresh wt. of *A. officinalis* were frozen in liquid nitrogen and lyophilized in a Jouan LP3 freeze dryer. The lyophilized material was transferred to EPR-type quartz tubes of 4 mm in diameter. Electron paramagnetic resonance measurements were performed at room temperature with a Bruker ELEXSYS X-band spectrometer (Bruker, Rheinstetten, Germany). The EPR spectra were recorded as first derivatives of microwave absorption. A microwave power of 2 mW and a magnetic field modulation of about 2 G were used for all the experiments to avoid signal saturation and deformation. EPR spectra of free radicals and Mn^2+^ ions were recorded in the magnetic field range of 3300–3360 G with 4096 data points. In order to determine the number of paramagnetic centers in the samples the spectra were double-integrated and compared with the intensity of the standard Al_2_O_3_:Cr^3+^ single crystal with a known spin concentration [16,31,32,33,34,35,36,37,56,57]. Before and after the first integration some background corrections of the spectra were made to obtain a reliable absorption signal before the second integration. Finally, EPR intensity data was calculated per 1 g of dry sample. It should be mentioned here that these tests had been made earlier and revealed that the process of freeze-drying (lyophilization) of the tissue did not cause free radical generation. Moreover, determination of the concentration of semiquinone radicals and the levels of Mn^2+^ ions using EPR was repeated three times, while plant material came from three independent cultures of *A. officinalis* infested by *B. asparagi*. Leaves (13–14 plants) from each experimental variant were collected to the EPR analysis. Each experiment was repeated three times.

### 4.4. Detection of Hydrogen Peroxide Release

Accumulation of H_2_O_2_ in one- and two-month-old asparagus leaves was detected by staining with a specific fluorescence agent, 2′,7′-dichlorofluorescein diacetate (DCFH-DA, Sigma-Aldrich, Poznań, Poland), following the methods of Małecka et al. [58] modified by Mai et al. [38]. DCFH-DA is non-fluorescent and can passively cross the membrane of live cells and it may be oxidized by H_2_O_2_ to a fluorescent dye detected by a laser confocal microscope. The fluorescence is proportional to the intracellular H_2_O_2_ level. The fresh leaves were submerged in 4 µM DCFH-DA dissolved for 12 h in 3 mM dimethylsulfoxide (DMSO) in 50 mM potassium phosphate buffer, pH 7.4. The leaves were washed twice with the loading buffer and then were observed under a confocal microscope (Zeiss LSM 510, Axioverd 200 M, Jena, Germany) with a filter set No. 10, excitation 488 nm, emission of 520 nm or more, and photographed using a digital camera (AxioCam, Zeiss, Jena, Germany). Microscope, laser and photomultiplier settings were held constant during the experiment in order to obtain comparable data. Images were analyzed using the LSM Image Browser software, version 4.2. Moreover, the observation of the generation of H_2_O_2_ under a confocal microscope were carried out on 4 leaves of 5 plants. Each experiment was repeated three times.

### 4.5. Detection of Superoxide Anion Radical Release

The release of O_2_^•−^ was detected using a fluorescent dye dihydroethidium (DHE, Sigma-Aldrich), described by Morkunas and Bednarski [35] and Mai et al. [16] with minor modifications. DHE is non-fluorescent and can passively cross the membrane of live cells, and it may be oxidized by O_2_^•−^ to a fluorescent dye detected by a laser confocal microscope. The fluorescence is proportional to the intracellular O_2_^•−^ level. The production of O_2_^•−^ in one- and two-month-old asparagus leaves was observed following the staining of surfaces with 100 µM DHE in 3 mM DMSO after immersing for 12 h at room temperature in the dark. After rinsing with the 100 µM CaCl_2_ solution, pH 4.75, leaf sections were observed using a Zeiss Axiovert 200M fluorescence microscope (model LSM 510, filter set No.9, excitation 450–490 nm, emission 520 nm or more, Zeiss, Jena, Germany), and photographed using a digital camera (AxioCam, Zeiss). An argon laser was used for excitation at 488 nm, with emission at 565–615 nm following background subtraction. All images were obtained at the same depth and were analyzed using the LSM Image Browser software, version 4.2. Moreover, the generation of O_2_^•−^ was observed under a confocal microscope on 4 leaves of 5 plants. Each experiment was repeated three times.

### 4.6. Extraction and Assay of Superoxide Dismutase Activity

Frozen leaves (0.50 g) were homogenized at 4 °C in 2.0 mL 50 mM sodium phosphate buffer (pH 7.0), containing 1.0 mM EDTA, 2% NaCl and 1% PVP (polyvinyl pyrrolidone) and centrifuged at 15,000× *g* for 15 min. The activity of SOD (EC 1.15.1.1) was spectrophotometrically assayed by measuring its ability to inhibit the photochemical reduction of nitro blue tetrazolium (NBT) according to Beauchamp and Fridovich [59], as described by Morkunas and Bednarski [35]. The 3 mL reaction mixture contained 50 mM sodium phosphate buffer (pH 7.8), 13 mM methionine, 75 mM NBT, 0.1 mM EDTA and 30 µL enzyme extract and 2 mM riboflavin (introduced to the reaction mixture as the last reagent). The reaction was started by switching on the light (two 15 W fluorescent lamps placed 30 cm below test tubes) and proceeded for 15 min. Samples without the enzymatic extract in the examined tests were selected so that the absorption difference between the blank and examined tests was about 50%. The amount of the enzyme that caused a 50% inhibition of NBT reduction was adopted as a unit of SOD activity. The activity of the enzyme was expressed as units per 1 mg of protein (U·mg^−1^ protein). The protein in the samples was determined according to Bradford [60] using bovine serum albumin (Sigma-Aldrich) as a standard. Moreover, leaves of 6–7 plants from each experimental variant were used to determine SOD activity. Each experiment was repeated three times.

### 4.7. Statistical Analysis

All determinations were performed in three independent experiments. Analysis of variance was applied to verify whether means from independent experiments within a given experimental variant were significant. The data was statistically analyzed using ANOVA, StatSoft, Inc. (2009, Poznań University of Life Sciences, Poznań, Poland), STATISTICA, version 9.0, available online: www.statsoft.com. Data shown in the figures represent means of triplicates for each variant and standard errors of mean (SE).

## 5. Conclusions

Defensive reactions in leaves of one- and two-month-old *A. officinalis* plants to *B. asparagi* were dependent on the intensity, duration of infestation and age of these plants. In leaves of one-month-old plant infested by aphids, an early higher generation of free radicals, both semiquinone and RFT, was recorded than in leaves of two-month-old plants infested by aphids. Therefore, the level of oxidative stress caused by *B. asparagi* was higher in leaves of one-month-old plants than in those of two-month-old plants. Strong fluctuations in Mn^2+^ ion levels versus time in *A. officinalis* leaves infested by *B. asparagi* indicate the involvement of these ions in the control of O_2_^•−^ production. The activity of SOD may be an important line of defense of two-month-old plants against oxidative stress caused by aphid attack.

## Figures and Tables

**Table 1:** Symptoms (96 hpi) on one- and two-month-old plants of *Asparagus officinalis* L. cv. Argenteuil caused by *Brachycorynella asparagi* (Mordvilko) at a varied population size, i.e., 10, 20, and 30 aphids per plant.

Symptoms	Variant		
	10	20	30
Aphids colonize the tops of the ferns	● ♦	● ♦	● ♦
Gently crimp the tops of the ferns	●	● ♦	● ♦
Shorter internodes		●	● ♦
Aphids colonize whole ferns		●	● ♦
The top of the ferns are twisted, with shortening internodes, “rosetting” and the plants turning yellow. The leaves are shortened and turned blue green			●

●—one-month old plants of asparagus; ♦—two-month old plants of asparagus.

## Abbreviations

EPR	electron paramagnetic resonance
fr. wt.	fresh weight
dry wt.	dry weight
g	gram
*g*-values	the factor describing energy levels of the unpaired electron for the paramagnetic centre in an external magnetic field
H_2_O_2_	hydrogen peroxide
h	hour
hpi	hours post infestation
Mn^2+^	manganese ions
ROS	reactive oxygen species
SOD	superoxide dismutase
O_2_^•−^	superoxide anion radical
